# Gut microbiome in paediatric short bowel syndrome: a systematic review and sequencing re-analysis

**DOI:** 10.1038/s41390-025-04083-0

**Published:** 2025-05-07

**Authors:** Jemma S. Cleminson, Gregory R. Young, David I. Campbell, Fiona Campbell, Andrew R. Gennery, Janet E. Berrington, Christopher J. Stewart

**Affiliations:** 1 Translational and Clinical Research Institute, Faculty of Medical Sciences, The Medical School, Newcastle University, Framlington Place, Newcastle upon Tyne, NE2 4HH UK; 2 Department of Paediatric Gastroenterology, Great North Children’s Hospital, Royal Victoria Infirmary, Newcastle upon Tyne, NE1 4LP UK; 3 Population Health Sciences Institute, Faculty of Medical Sciences The Medical School Newcastle University Framlington Place, Newcastle upon Tyne, NE2 4HH UK; 4 Newcastle University Institute of Population Health Sciences The Catalyst Room 3.12, 3 Science Square Newcastle Helix, Newcastle Upon Tyne, NE4 5TG UK; 5 Department of Paediatric Immunology and HSCT, Great North Children’s Hospital, Royal Victoria Infirmary, Newcastle upon Tyne, NE1 4LP UK; 6 Department of Neonatology, Ward 35, Level 4, Leazes Wing, Royal Victoria Infirmary, Newcastle upon Tyne, Tyne and Wear NE1 4LP UK

## Abstract

- Children with short bowel syndrome depend on parenteral nutrition, which carries significant risks.
- Short bowel syndrome patients show reduced gut microbial diversity, increased inflammation-associated bacteria, and fewer beneficial bacteria.
- This is the first systematic review and meta-analysis examining the gut microbiome in children with short bowel syndrome.
- The review demonstrated significantly lower bacterial diversity and richness in children with short bowel syndrome, regardless of achievement of intestinal autonomy. Diversity and richness were greater in children who achieved intestinal autonomy than those on parenteral nutrition, though not statistically significant.
- Larger studies adjusting for confounding factors may identify future therapeutic strategies.

## Introduction

Paediatric intestinal failure (IF) is defined as the “…the reduction of functional intestinal mass below that which can sustain life, resulting in dependence on supplemental parenteral support for a minimum of 60 days…”.^1^ Short bowel syndrome (SBS), often resulting from extensive bowel resection, is the leading cause of paediatric IF (SBS-IF).^2^ Parenteral nutrition (PN) is lifesaving but expensive and associated with important complications, notably central line-associated bloodstream infection (CLABSI), intestinal failure-associated liver disease (IFALD), thrombosis and mortality.^2^ To achieve intestinal autonomy (IA), structural and functional adaptations occur to the remaining bowel to enhance absorptive capacity, allowing for eventual discontinuation of PN, a process that can take years^3,4^ Predicting when IA will occur is difficult due to the rarity and heterogeneity of the condition.

Recent studies link the gut microbiome with gut physiology.^5^ Altered gut microbial diversity and composition are linked to disease.^5^ Studies show children with SBS have a distinct gut microbiome compared to healthy peers, characterised by reduced microbial diversity, increased proportional abundance of inflammation-associated bacteria (e.g., Proteobacteria, especially Enterobacteriaceae), and reduced ‘beneficial’ bacteria (e.g., Bacteroidetes).^6–18^ SBS patients also do not undergo age related maturational progression of gut microbiome as seen in healthy children.^11^

Understanding microbiome changes in SBS may guide future treatments, but current studies have relatively small sample sizes (*n* = 7 to 25).^7,12^ This systematic review and meta-analysis is the first to examine the gut microbiome in children with SBS.

## Aims and objectives

To compare the gut microbial diversity and composition between children with SBS on PN to those that have achieved intestinal autonomy and to healthy controls.

## Methods

We searched Ovid MEDLINE (1946 to November Week 5 2023), EMBASE (1974 to 2023 December 11), Cochrane Library (to 12/12/2023) and clinical trial registries (World Health Organisation’s International Clinical Trials Registry Platform, ClinicalTrials.gov, and the ISRCTN Registry).

The search strategy included mesh and keywords (supp.) for studies that met the following criteria:

- Population: Children with SBS < 18 years
- Exposure: PN
- Comparator:
  1. Children with SBS weaned off PN
  2. Healthy controls <18 years
- Outcome: Gut bacterial diversity and composition, measured by 16S rRNA gene sequencing of the V3 or V3/4 hypervariable regions.
- Study design: Case control or cohort

We performed reference searching of included studies. We included studies exclusively sequencing stool microbes by targeting the V3 or V3/4 hypervariable regions of the 16S rRNA gene, being the modal form of microbiome interrogation, to avoid issues arising due to the impact of 16S rRNA target region on the estimated proportions of taxa.^19^ We excluded studies that sequenced other hypervariable regions of the 16S rRNA gene (*n* = 5), performed shotgun metagenomic sequencing (*n* = 1), or where no sequencing data was available (*n* = 7). We contacted the corresponding authors for these studies via email. Among those who responded, one reported that the data was destroyed, and another was invited to share data but ultimately did not provide it. We excluded non-faecal samples and those samples taken when the participant was on antibiotics.

Two reviewers independently performed title, abstract and full text screening. One reviewer extracted data. A second reviewer verified data. Raw sequencing data were downloaded from repositories described in the originating studies. Final included studies were assessed for risk of bias using the Newcastle Ottawa Score^20^(Supplementary Table 1). We conducted the original search in November 2023 and re-ran it before submission to ensure the meta-analysis remained up to date with published literature. No further studies that would meet inclusion criteria were identified.

### Sequence processing and analysis

Sequencing data were processed using Qiime2.^21^ Studies targeting similar hyper-variable regions were processed together.^22^ Operational taxonomic unit (OTU) relative abundance per sample was calculated then OTUs were merged at the genus level and samples were rarefied to 3000 reads. Samples with reported current antibiotic exposure were excluded.

Analyses were performed in R studio^23^ using the phyloseq^24^ and vegan packages.^25^ Comparisons between continuous and categorical variables were made with Kruskal-Walis rank-sum and Fisher’s exact test, respectively. Where no definition for IA was given, participants with SBS that were no longer dependent on PN were assumed to have achieved IA. Associations between SBS, PN and alpha diversity (Shannon diversity and taxonomic richness) were interrogated with generalised linear mixed models, including participant as a random and originating study as a fixed effect. SBS status influence on microbiota compositions (Bray-Curtis dissimilarity) were assessed with PERMANOVA, including only one sample per participant based on priorities of ‘on > off PN’ and ‘earlier > later samples’. MaAsLin2 was used to identify differentially abundant taxonomic features between groups.

## Results

We identified 1581 articles after deduplication (Supplementary Fig. 1). Fifteen full texts were eligible for inclusion. A further two studies were identified by searching citations of included studies.^9,26^ Studies with unavailable sequencing data (*n* = 7) or employing incommensurable sequencing strategies (*n* = 5) were excluded (Supplementary Table 2). Five studies were finally included.^7,9,10,14,27^ There were 3 cohort and 2 case-control studies. The number of children included in the studies ranged from 5 to 25 with sequencing data downloaded for all. Three sequenced the V3/V4 region^7,9,14^ and two the V4 region^10,27^ (Table 1).

**Table 1:** Included studies.

Author, Year	Reference
Engstrand Lilja,^9^	Engstrand Lilja H, Wefer H, Nyström N, Finkel Y, Engstrand L. Intestinal dysbiosis in children with short bowel syndrome is associated with impaired outcome. Microbiome. 2015;3:18.
Piper,^10^	Piper HG, Fan D, Coughlin LA, Ho EX, McDaniel MM, Channabasappa N, Kim J, Kim M, Zhan X, Xie Y, Koh AY. Severe Gut Microbiota Dysbiosis Is Associated With Poor Growth in Patients With Short Bowel Syndrome. JPEN J Parenter Enteral Nutr. 2017 Sep;41(7):1202-1212.
Zeichner,^7^	Zeichner SL, Mongodin EF, Hittle L, Huang S-H, Torres C. The bacterial communities of the small intestine and stool in children with short bowel syndrome. PLOS ONE. 2019;14(5):e0215351
Phyo,^14^	Phyo LY, Singkhamanan K, Laochareonsuk W, Surachat K, Phutong N, Boonsanit K, et al. Faecal microbiome alterations in paediatric patients with short bowel syndrome receiving a rotating cycle of gastrointestinal prophylactic antibiotics. Paediatric Surgery International. 2021;37(10):1371-81.
Neelis, 2022	Neelis EG, de Koning BAE, Hulst JM, Papadopoulou R, Kerbiriou C, Rings EHHM, Wijnen RMH, Nichols B, Gerasimidis K. Gut microbiota and its diet-related activity in children with intestinal failure receiving long-term parenteral nutrition. JPEN J Parenter Enteral Nutr. 2022 Mar;46(3):693-708. 10.1002/jpen.2188. Epub 2021 Aug 25.

Sequencing data was downloaded for 234 total samples from 133 participants. Only one study (Phyo et al.^14^) included sufficiently granular antibiotic data to facilitate inclusion of this variable in analysis. Re-analysis of antibiotic impact in the context of SBS would simply replicate those already presented by Phyo et al. in their original manuscript. As such, all samples with known antibiotic exposure were excluded. Removal of participants with functional IF, jejunal samples, and faecal samples taken from participants with reported current antibiotic exposure yielded 129 faecal samples from 95 participants (Supplementary Tables 3, 4). Sequencing depth (*p* < 0.001)(Supplementary Fig. 2) and microbiota compositions (*p* = 0.001, R^2^ = 0.28)(Supplementary Fig. 3) varied significantly between studies. All samples were therefore rarefied at 3000 reads(Supplementary Fig. 4). Five samples with fewer than 3000 reads were discarded. Microbiota compositions were most similar in repeated samples from single participants (R^2^ = 0.55; *p* = 0.001). We therefore selected the earliest sample per participant for analysis. Following de-duplication, 94 faecal samples from 94 participants were included in analysis.

**Fig. 1:**
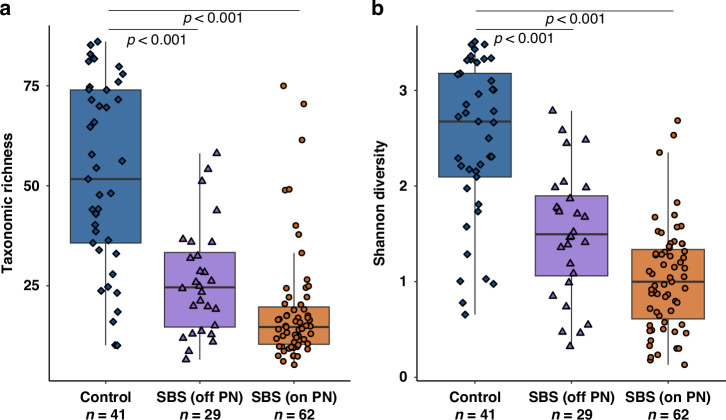
Alpha diversity differences between healthy control participants and those with SBS on PN and having achieved intestinal autonomy. The centre of boxplots of Shannon diversity (**a**) and taxonomic richness (**b**) illustrate the mean for each group. Boxes extend to the interquartile range and whiskers extend to the full range of data (excluding outliers). Significant differences (*p* < 0.05) are highlighted above the comparison.

Bacterial diversity (1.23 ± 0.11) and richness (27.8 ± 3.07) were significantly lower in participants with SBS on PN than healthy controls (HC) (Diversity: 2.32 ± 0.12; *p* < 0.001 | Richness: 47.4 ± 2.54; *p* < 0.001). Likewise, participants with SBS who achieved IA had significantly lower bacterial diversity (1.49 ± 0.15; *p* < 0.001) and richness (24.6 ± 2.19; *p* < 0.001) than HCs. No significant differences were observed between participants achieving IA and those still on PN (Fig. 1).

**Fig. 2:**
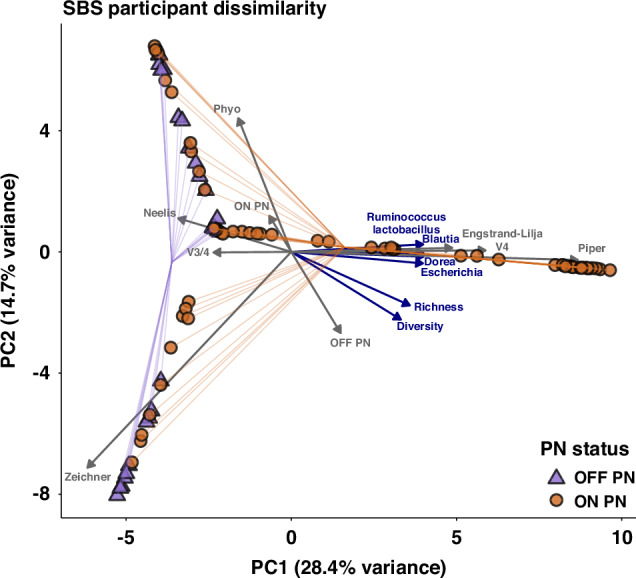
Principal coordinate analysis (PCoA) plot visualising gut microbiome dissimilarity among SBS participants. The plot reveals differences in microbiome composition based on PN dependence, microbial diversity, and key bacterial associations. Each point represents an individual sample. Circles represent samples from participants with IF-SBS who are dependent on PN, and triangles indicate those who have achieved IA. Principal components are calculated from weighted Bray-Curtis dissimilarity. Points are joined to a centroid, representing the average microbiota composition of participants with that PN status. Vector arrows illustrate the impact of study (grey) and microbial (blue) metrics on microbial community composition. Only genera identified as differential between PN status with MaAsLin2 (*p* < 0.05) are plotted as microbial metrics.

While associated with microbiota composition when assessed in isolation (R^2^ = 0.02; *p* = 0.01), PN status was not significantly associated with microbiota composition (R^2^ = 0.08; *p* = 0.57) when originating study (R^2^ = 0.21; *p* = 0.001) was included as a co-variate (Fig. 2).

Proportional abundances of *Dorea* (q = 0.002)*, Ruminococcus* (q = 0.04) and *Blautia* (*p* = 0.004) were significantly lower in SBS on PN than not. In contrast, 17 taxa (including all those associated with PN status) were differentially abundant between originating studies included here (Supplementary Table 5).

### Risk of bias in included studies

All studies were of fair quality, with potential selection bias due to a lack of descriptions of participant selection, unmatched controls, and unadjusted confounding factors. Seven studies were excluded due to unavailable sequencing data and four were excluded for sequencing incompatible hypervariable regions, introducing reporting bias. Two included studies did not include participants that had achieved IA, thus we cannot discount the differences in study methodology from describing the differential abundances of genera in children with IF-SBS on PN. Additionally, studies varied in terms of geography, diet, medical practices, and participant selection, all of which may have influenced microbial composition.

## Discussion

This systematic review aimed to evaluate the gut microbiome in children with SBS. Previous studies are underpowered and showed varying study design and inconsistent results, but describe reduced diversity, increased abundances of inflammation-associated bacteria, and decreased genera associated with health ‘benefit’ in these patients(8)^11^(8–15).^9^ The current meta-analysis including 20 children with IA, 33 still on PN, and 41 HCs, demonstrated significantly lower bacterial diversity and richness in SBS children regardless of whether they had achieved IA. Diversity and richness were greater in SBS patients who had achieved IA than those on PN, though this was not statistically significant.

Analysis of specific bacterial genera revealed that *Dorea, Ruminococcus*, and *Blautia*, of the commensal Lachnospaceae family were relatively less abundant in SBS patients on PN(24)than those with IA. These findings suggest that the gut microbiome of SBS children on PN differs markedly from that of HCs, and becomes more similar to HCs as patients progress towards IA. This could be attributed to factors such as cumulative lifetime antibiotic exposure (indications, class, dosing) and small intestine bacterial overgrowth,^7,11,27^ which limits availability of dietary fibres that support beneficial short-chain fatty acid (SCFA)-producing bacteria.^8^ SCFAs play a key role in gut epithelial health and energy metabolism, and their reduction is associated with mucosal inflammation.^28–30^ Enteral nutrition promotes microbial diversity, providing essential substrates for beneficial bacteria, stimulating gut motility, and creating an environment conducive to a more diverse microbiome.^31^ Considering these complex interactions, this transition may be the result of, or indeed promote, IA.

This study represents the first meta-analysis of the gut microbiome in paediatric SBS, pooling data from 94 children (*n* = 33 with SBS on PN, *n* = 20 IA, and *n* = 41 HCs). This methodology increases statistical power, providing a more reliable estimate of effects and enabling results to be controlled for inconsistencies across studies. However, these findings may be limited by the risk of methodological selection bias, lack of matched controls, and unadjusted confounders. Twelve studies were excluded from the presented analysis due to inaccessibility of data or targeting incomparable 16S rRNA variable regions. Utilising untargeted metagenomic or full-length 16S rRNA sequencing methods would maximise the utility and comparability of any future studies in this area.

While this systematic review provides valuable insights into the gut microbiome in paediatric SBS, further research is needed. Prospective cohort studies could further clarify gut microbiome dynamic changes during SBS treatment, particularly in predicting IA. Future studies should aim for larger cohort and, ideally, matched controls, controlling for confounding factors such as antibiotic use, age, and dietary intake. Achieving true independence in matched controls is challenging outside of randomised controlled trials, as observational data are limited by residual confounding factors and selection biases. However, a deeper understanding of the gut microbiome in SBS may drive research into microbiome-modulating therapies ultimately improving clinical management strategies for this complex condition. Such approaches could include specific enteral nutrition strategies, selected pre- and probiotic use, short-chain fatty acid supplementation, judicious use of antibiotics, and faecal microbiome transplantation.

## Data availablity

The datasets generated during and/or analysed during the current study are available from the corresponding author on reasonable request.

## Supplementary information

Supplementary material

## Study Registration

The study protocol was registered with OSF: registration number osf.io/385rj, on 5^th^ April 2024 10.17605/OSF.IO/5PHU7.
