# Supplementary material for: Gut microbiome in paediatric short bowel syndrome: a systematic review and sequencing re-analysis
Source: Pediatr Res. 2025 May 7;98(6):2082–6. doi: 10.1038/s41390-025-04083-0 (PMC12811130; doi:10.1038/s41390-025-04083-0)
Supplement: Supplementary file 1 — Supplementary material [file 41390_2025_4083_MOESM1_ESM.pdf]

Gut microbiome in paediatric short bowel syndrome: A systematic review and sequencing re-analysis
Supplementary information

Search Strategy 2

Supplementary figure S1 – PRISMA 2020 flow diagram 4

Papers identified from literature review 5

Supplementary table S1 – Newcastle Ottawa Score of included studies 6

Supplementary table S2 – Characteristics of excluded studies 7

Supplementary table S3 - Studies, Participants, samples and bacterial sequencing reads included in systematic review and meta-analysis 9

Supplementary table S4- Participant demographics for deduplicated samples included in systematic review and meta-analysis 10

Supplementary figure S2 - Sequencing library sizes are significantly different between originating studies included in this systematic review and meta-analysis 12

Supplementary figure S3 – Microbiota compositions are significantly different between originating studies included in this systematic review and meta-analysis 13

Supplementary figure S4 – Rarefaction depth of 3000 reads is sufficient to characterise microbiota of samples included in this systematic review and meta-analysis 14

Supplementary table S5 – MaAsLin2 results identify differentially abundant bacterial genera between participants on and off PN as well as between originating studies in this systematic review and meta-analysis 15

Search Strategy

Ovid MEDLINE(R) <1946 to November Week 5 2023>

1	Short Bowel Syndrome/	3372
2	short gut.mp.	459
3	intestinal failure.mp.	2290
4	exp Parenteral Nutrition/	25489
5	microbiota/ or gastrointestinal microbiome/	73372
6	Microbiota/	37402
7	microbiota.mp.	89960
8	exp Bacteria/	1537975
9	microbiome.mp.	67109
10	RNA, Ribosomal, 16S/67764	
11	Metagenomics/ or Metagenome/ or metagenom*.mp.	26960
12	5 or 7 or 8 or 9 or 10 or 11	1617880
13	exp child/	2174488
14	p?ediatr*.mp.	459035
15	infant*.mp.	1363292
16	toddler*.mp.	12729
17	adolescen*.mp.	2268636
18	teen*.mp.	31923
19	Premature Birth/	21972
20	13 or 14 or 15 or 16 or 17 or 18 or 19	4169913
21	animal*.mp.	7473321
22	human*.mp.	22040716
23	21 not 22	4850524
24	1 or 2 or 3 or 4	28560
25	12 and 20 and 24	272
26	25 not 23	264

Embase <1974 to 2023 December 11>

1	Short Bowel Syndrome/	7600
2	short gut.mp.	901
3	intestinal failure.mp.	5216
4	exp Parenteral Nutrition/	58031
5	microbiota/ or gastrointestinal microbiome/	111196
6	Microflora/	32787
7	microbiota.mp.	114772
8	exp Bacterium/	1890836
9	microbiome.mp.	80198
10	exp RNA 16S/	100044
11	Metagenomics/ or Metagenome/ or metagenom*.mp.	42990
12	5 or 7 or 8 or 9 or 10 or 11	2014018
13	exp child/	3133808
14	p?ediatr*.mp.	842627
15	infant*.mp.	1020949
16	toddler*.mp.	19899
17	adolescen*.mp.	1923184

18	teen*.mp.	50803
19	exp prematurity/	129057
20	13 or 14 or 15 or 16 or 17 or 18 or 19	4475816
21	animal*.mp.	6665734
22	human*.mp.	26933380
23	21 not 22	4648681
24	1 or 2 or 3 or 4	64094
25	12 and 20 and 24	1394
26	25 not 23	1349

Cochrane Library
Search Name: SBS scoping review.
Date Run: 12/12/2023 16:11:36

ID	Search	Hits
#1	paediatric* OR Pediatric* OR child* OR infant* OR toddler* OR adolescent* OR teen*	359293
#2	"microbiota" OR "gastrointestinal microbiome" OR "microbiome" OR "bacteria"	25159
#3	"short bowel syndrome" OR "short gut" OR "intestinal failure" OR "parenteral nutrition"	5053
#4	#1 AND #2 AND #3	133

Supplementary figure S1 – PRISMA 2020 flow diagram

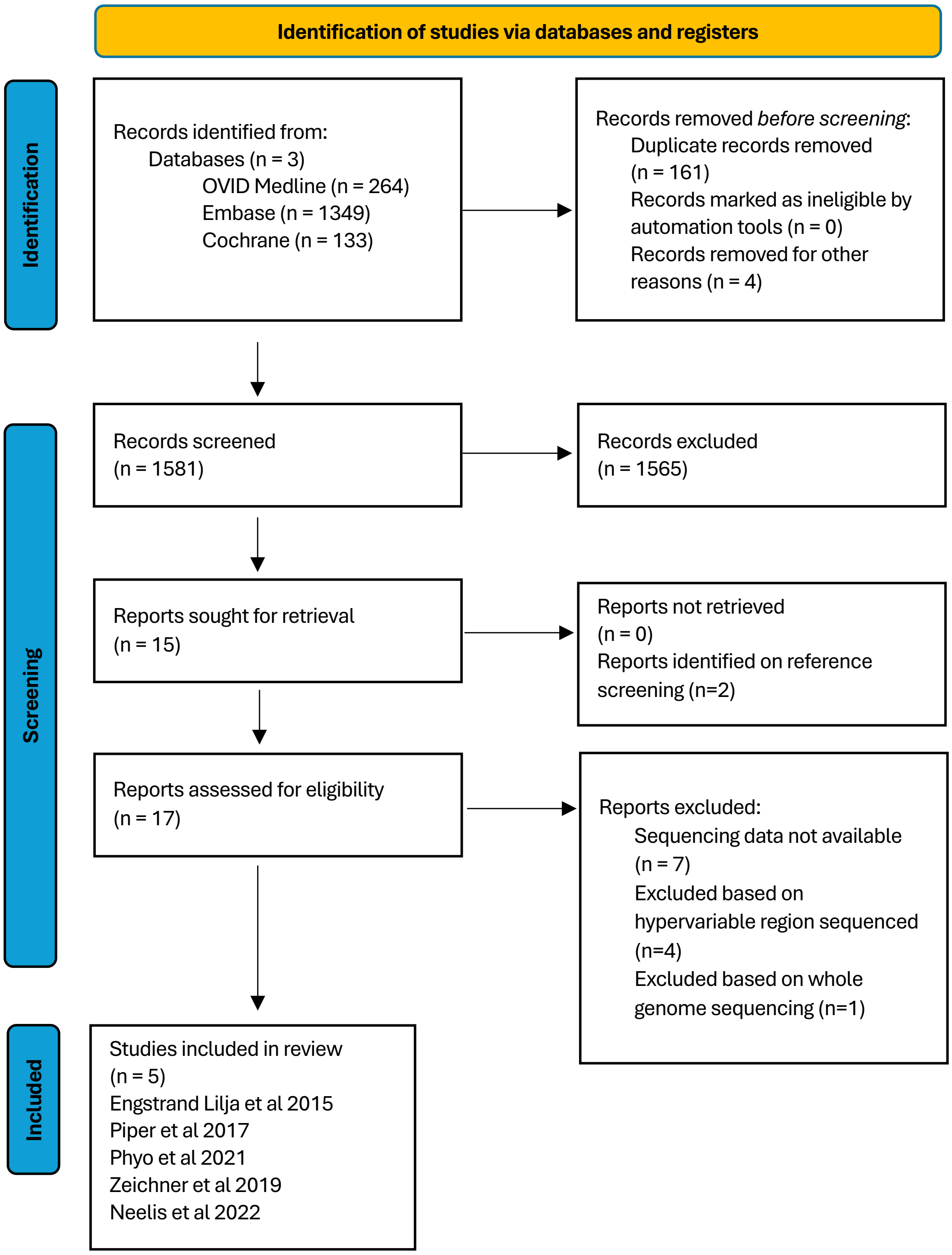

Papers identified from literature review

1. Zeichner SL, Mongodin EF, Hittle L, Huang S-H, Torres C. The bacterial communities of the small intestine and stool in children with short bowel syndrome. PLOS ONE. 2019;14(5):e0215351
2. Piper HG, Fan D, Coughlin LA, Ho EX, McDaniel MM, Channabasappa N, et al. Severe Gut Microbiota Dysbiosis Is Associated With Poor Growth in Patients With Short Bowel Syndrome.1.
3. Neelis EG, de Koning BAE, Hulst JM, Papadopoulou R, Kerbiriou C, Rings E, et al. Gut microbiota and its diet-related activity in children with intestinal failure receiving long-term parenteral nutrition.1.
4. Korpela K, Mutanen A, Salonen A, Savilahti E, de Vos WM, Pakarinen MP. Intestinal Microbiota Signatures Associated With Histological Liver Steatosis in Pediatric-Onset Intestinal Failure.1.
5. Dowhaniuk JK, Szamosi J, Chorlton S, Owens J, Mileski H, Clause RF, et al. Starving the Gut: A Deficit of Butyrate in the Intestinal Ecosystem of Children With Intestinal Failure.1.
6. Piper HG. Intestinal microbiota in short bowel syndrome. Semin Pediatr Surg. 2018;27(4):223-8.
7. Thänert R, Thänert A, Ou J, Bajinting A, Burnham C-AD, Engelstad HJ, et al. Antibiotic-driven intestinal dysbiosis in pediatric short bowel syndrome is associated with persistently altered microbiome functions and gut-derived bloodstream infections. Gut Microbes. 2021;13(1):1940792.
8. Zhang T, Wang Y, Yan W, Lu L, Tao Y, Jia J, et al. Microbial alteration of small bowel stoma effluents and colonic feces in infants with short bowel syndrome. J Pediatr Surg. 2019.
9. Piper HG, Coughlin LA, Nguyen V, Channabasappa N, Koh AY. A comparison of small bowel and fecal microbiota in children with short bowel syndrome. Journal of pediatric surgery : official journal of [the] Surgical Section of the American Academy of Pediatrics, British Association of Paediatric Surgeons, American Pediatric Surgical Association, Canadian Association of Pediatric Surgeons. 2020;55(5):878-82.
10. Phyo LY, Singkhamanan K, Laochareonsuk W, Surachat K, Phutong N, Boonsanit K, et al. Fecal microbiome alterations in pediatric patients with short bowel syndrome receiving a rotating cycle of gastrointestinal prophylactic antibiotics. Pediatric Surgery International. 2021;37(10):1371-81.
11. Davidovics ZH, Carter BA, Luna RA, Hollister EB, Shulman RJ, Versalovic J. The Fecal Microbiome in Pediatric Patients With Short Bowel Syndrome. Journal of Parenteral and Enteral Nutrition. 2016;40(8):1106-13.
12. Wang P, Wang Y, Lu L, Yan W, Tao Y, Zhou K, et al. Alterations in intestinal microbiota relate to intestinal failure-associated liver disease and central line infections. Journal of pediatric surgery : official journal of [the] Surgical Section of the American Academy of Pediatrics, British Association of Paediatric Surgeons, American Pediatric Surgical Association, Canadian Association of Pediatric Surgeons. 2017;52(8):1318-26.
13. Engelstad HJ, Barron L, Moen J, Wylie TN, Wylie K, Rubin DC, et al. Remnant Small Bowel Length in Pediatric Short Bowel Syndrome and the Correlation with Intestinal Dysbiosis and Linear Growth. Journal of the American College of Surgeons. 2018;227(4):439-49.
14. Talathi S, Wilkinson L, Meloni K, Shroyer M, Eipers P, Van Der Pol WJ, et al. Scheduled Empiric Antibiotics May Alter the Gut Microbiome and Nutrition Outcomes in Pediatric Intestinal Failure. Nutr Clin Pract. 2021;36(6):1230-9.
15. Talathi S, Wilkinson L, Meloni K, Shroyer M, Zhang L, Ding Z, et al. Factors affecting the Gut Microbiome in Pediatric Intestinal Failure. J Pediatr Gastroenterol Nutr. 2023
16. Budinska E, Gojda J, Heczkova M, Bratova M, Dankova H, Wohl P, et al. Microbiome and Metabolome Profiles Associated With Different Types of Short Bowel Syndrome: Implications for Treatment. Journal of Parenteral and Enteral Nutrition. 2020;44(1):105-18. (identified from reference searching)
17. Engstrand Lilja H, Wefer H, Nyström N, Finkel Y, Engstrand L. Intestinal dysbiosis in children with short bowel syndrome is associated with impaired outcome. Microbiome. 2015;3:18. (identified from references searching)

Supplementary table S1 – Newcastle Ottawa Score of included studies

Study	Sample size	Limitations	Selection	Comparability	Exposure	Total	Quality
Engstrand Lilja 2015	18	No definition of SBS, potential selection bias; no matching	2	0	3	5	Fair
Piper 2017	11	HCs from different community; potential selection bias; no matching	3	0	2	5	Fair
Zeichner 2019	29	Potential selection bias; no matching; hospital controls (children needing endoscopy)	2	0	3	5	Fair
Phyo 2021	20	Potential selection bias; no matching; hospital controls (children in outpatient clinics)	2	0	2	4	Fair
Neelis 2022	33	Potential selection bias;	3	0	2	5	Fair

Supplementary table S2 – Characteristics of excluded studies

Lead author	Year	Reason for exclusion
Korpela(1)	2017	Incompatible hypervariable regions (V1/V6)
Davidovics (2)	2016	Incompatible hypervariable regions (V3/V5)
Wang(3)	2017	Incompatible hypervariable regions (V4/V5)
Budinska(4)	2020	Incompatible hypervariable regions (V1-V3)
Thanert(5)	2021	Whole genome sequencing
Engelstad(6)	2018	Unavailable sequencing data
Dowhaniuk (7)	2020	Unavailable sequencing data
Piper(8)	2020	Unavailable sequencing data
Piper(9)	2020	Unavailable sequencing data
Zhang (10)	2020	Unavailable sequencing data
Talathi(11)	2021	Unavailable sequencing data
Talathi(12)	2023	Unavailable sequencing data

1. Korpela K, Mutanen A, Salonen A, Savilahti E, de Vos WM, Pakarinen MP. Intestinal Microbiota Signatures Associated With Histological Liver Steatosis in Pediatric-Onset Intestinal Failure. JPEN J Parenter Enteral Nutr. 2017;41(2):238-48.
2. Davidovics ZH, Carter BA, Luna RA, Hollister EB, Shulman RJ, Versalovic J. The Fecal Microbiome in Pediatric Patients With Short Bowel Syndrome. Journal of Parenteral and Enteral Nutrition. 2016;40(8):1106-13.
3. Wang P, Wang Y, Lu L, Yan W, Tao Y, Zhou K, et al. Alterations in intestinal microbiota relate to intestinal failure-associated liver disease and central line infections. Journal of pediatric surgery : official journal of [the] Surgical Section of the American Academy of Pediatrics, British Association of Paediatric Surgeons, American Pediatric Surgical Association, Canadian Association of Pediatric Surgeons. 2017;52(8):1318-26.
4. Budinska E, Gojda J, Heczkova M, Bratova M, Dankova H, Wohl P, et al. Microbiome and Metabolome Profiles Associated With Different Types of Short Bowel Syndrome: Implications for Treatment. Journal of Parenteral and Enteral Nutrition. 2020;44(1):105-18.
5. Thanert R, Thanert A, Ou J, Bajinting A, Burnham CAD, Engelstad HJ, et al. Antibiotic-driven intestinal dysbiosis in pediatric short bowel syndrome is associated with persistently altered microbiome functions and gut-derived bloodstream infections. 2021(1):no pagination.
6. Engelstad HJ, Barron L, Moen J, Wylie TN, Wylie K, Rubin DC, et al. Remnant Small Bowel Length in Pediatric Short Bowel Syndrome and the Correlation with Intestinal Dysbiosis and Linear Growth. Journal of the American College of Surgeons. 2018;227(4):439-49.
7. Dowhaniuk JK, Szamosi J, Chorlton S, Owens J, Mileski H, Clause RF, et al. Starving the Gut: A Deficit of Butyrate in the Intestinal Ecosystem of Children With Intestinal Failure. Journal of Parenteral and Enteral Nutrition. 2020;44(6):1112-23.
8. Piper HG, Coughlin LA, Nguyen V, Channabasappa N, Koh AY. A comparison of small bowel and fecal microbiota in children with short bowel syndrome. Journal of pediatric surgery : official journal of [the] Surgical Section of the American Academy of Pediatrics, British Association of Paediatric Surgeons, American Pediatric Surgical Association, Canadian Association of Pediatric Surgeons. 2020;55(5):878-82.

9. Piper HG, Coughlin LA, Hussain S, Nguyen V, Channabasappa N, Koh AY. The Impact of Lactobacillus Probiotics on the Gut Microbiota in Children With Short Bowel Syndrome. J Surg Res. 2020;251:112-8.
10. Zhang T, Wang Y, Yan W, Lu L, Tao Y, Jia J, et al. Microbial alteration of small bowel stoma effluents and colonic feces in infants with short bowel syndrome. J Pediatr Surg. 2020;55(7):1366-72.
11. Talathi S, Wilkinson L, Meloni K, Shroyer M, Eipers P, Van Der Pol WJ, et al. Scheduled Empiric Antibiotics May Alter the Gut Microbiome and Nutrition Outcomes in Pediatric Intestinal Failure. Nutr Clin Pract. 2021;36(6):1230-9.
12. Talathi S, Wilkinson L, Meloni K, Shroyer M, Zhang L, Ding Z, et al. Factors affecting the Gut Microbiome in Pediatric Intestinal Failure. J Pediatr Gastroenterol Nutr. 2023.

Supplementary table S3 - Studies, Participants, samples and bacterial sequencing reads included in systematic review and meta-analysis

	TOTAL COHORT	NO AbX	FAECAL ONLY	>3k READS	Deduplicated
Studies	5	5	5	5	5
Participants	133	133	107	95	94
Samples	234	172	146	129	94
Total reads				10,511,909	7,621,385
Median reads				37,770	40,440
IQR				22,599 - 95,504	25,365 – 96,202

Abx = antibiotics; IQR = interquartile range

Supplementary table S4- Participant demographics for deduplicated samples included in systematic review and meta-analysis

	Included Studies					
	Piper	*Engstrand-Lilja*	*Neelis*	*Zeichner*	*Phyo*	*All studies*
**Participants (samples)**						
Total	5 (18)	15 (15)	29 (29)	25 (26)	20 (42)	94 (129)
Healthy Control	0 (0)	7 (7)	23 (23)	4 (4)	7 (7)	41 (41)
Short Bowel Syndrome	5(18)	8 (8)	6 (6)	21 (22)	13 (35)	53 (87)
Parenteral Nutrition	3 (8)	2 (2)	6 (6)	9 (10)	13 (35)	33 (61)
Intestinal Rehabilitation	2 (10)	6 (6)	0 (0)	12 (12)	0 (0)	20 (28)
**Study Characteristics**						
doi	https://doi.org/10.1177/0148607116658762	https://doi.org/10.1186/s40168-015-0084-7	https://doi.org/10.1002/jpen.2188	https://doi.org/10.1371/journal.pone.0215351	https://doi.org/10.1007/s00383-021-04948-5	
Year	2017	2015	2021	2019	2021	
Location	USA	Sweden	Netherlands	USA	Thailand	
Design	Cohort	Case-control	Cohort	Case-control	Cohort	
Definition of SBS / inclusion criteria	Children with SBS (dependent on PN for at least 3 months secondary to small intestinal loss)	Children with SBS diagnosed in neonatal period	Surgical IF patients included in metaanalysis. SBS was defined as a resection of >70% of the small intestine and/or a remaining length of the small intestine (measured from the ligament of Treitz onward) of <50 cm in preterm infants or <75 cm in term infants and	Surgical resection of NEC or a congenital anomaly such as gastroschisis or intestinal atresia, stable at home for > 4 weeks	Children who underwent small intestinal resection with remaining small intestine < 100 cm and needed PN > 6 months	

			stable while receiving home PN (>3 months). Surgical IF also included patients with SBS and patients who had a minor resection of the small bowel but did not fulfil the criteria for SBS.			
Definition of IR	NR	NR	NA	NR	NA	
Variable region sequenced	V4	V3/4	V4	V3/4	V3/4	
**Age at sampling** (Median[IQR])						
Healthy Control	NA	10.0 [4.5-11.0]	6.6 [2.5-9.6]	7.0 [4.5-9.3]	NA	6.6 [3.0-10.0
Short Bowel Syndrome	NA	3.0 [2.8-4.5]	6.1 [4.3-7.4]	4.5 [3.0-6.0]	NA	4.0 [3.0-6.0]
**Sex** (Male/Female/NA)						
Healthy Control	0/0/0	0/0/7	10/13/0	1/3/0	0/0/7	11/16/14
Short Bowel Syndrome	0/0/5	0/0/8	3/3/0	5/16/0'	0/0/13	8/19/26

SBS = Short Bowel Syndrome; PN = Parenteral Nutrition; IF = Intestinal Rehabilitation; NEC = Necrotising Enterocolitis; NR = Not relevant (study includes no participants achieved IR); NA = Data not available; V = 16S rRNA gene Variable region; IQR = interquartile range

<u>Supplementary figure S2 - Sequencing library sizes are significantly different between originating studies included in this systematic review and meta-analysis</u>

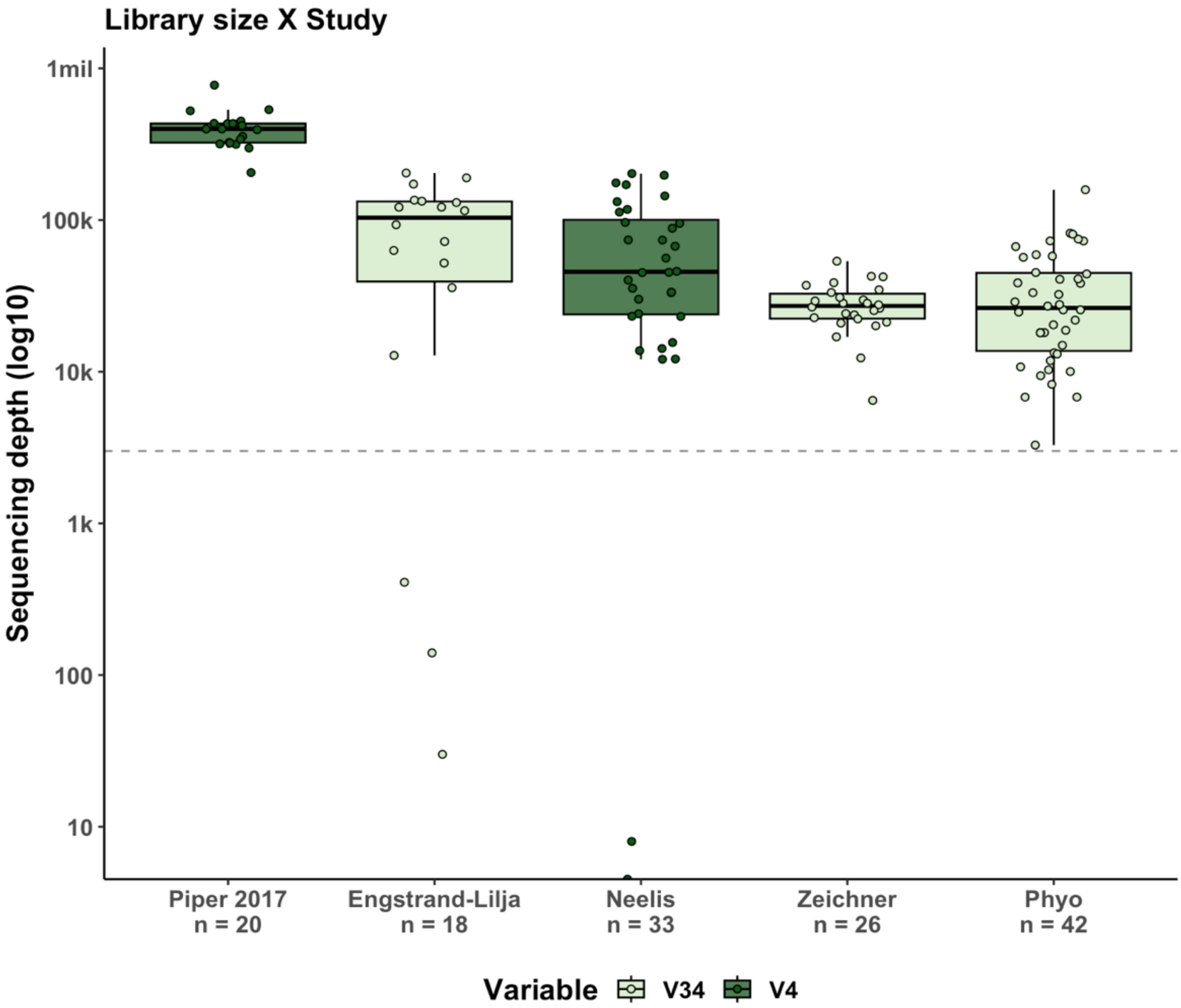

Sequencing library size of reads mapping to bacteria across each of the five studies included in this analysis. Median values per study are depicted with black lines. Boxes (coloured by hypervariable region targeted for sequencing) represent the interquartile range. Whiskers extend to the full range of data (excluding outliers). Points represent individual samples. Dotted line intersecting y-axis represents the rarefaction cut-off of 3000 reads. All samples below this threshold (5) are culled from analysis.

Supplementary figure S3 – Microbiota compositions are significantly different between originating studies included in this systematic review and meta-analysis

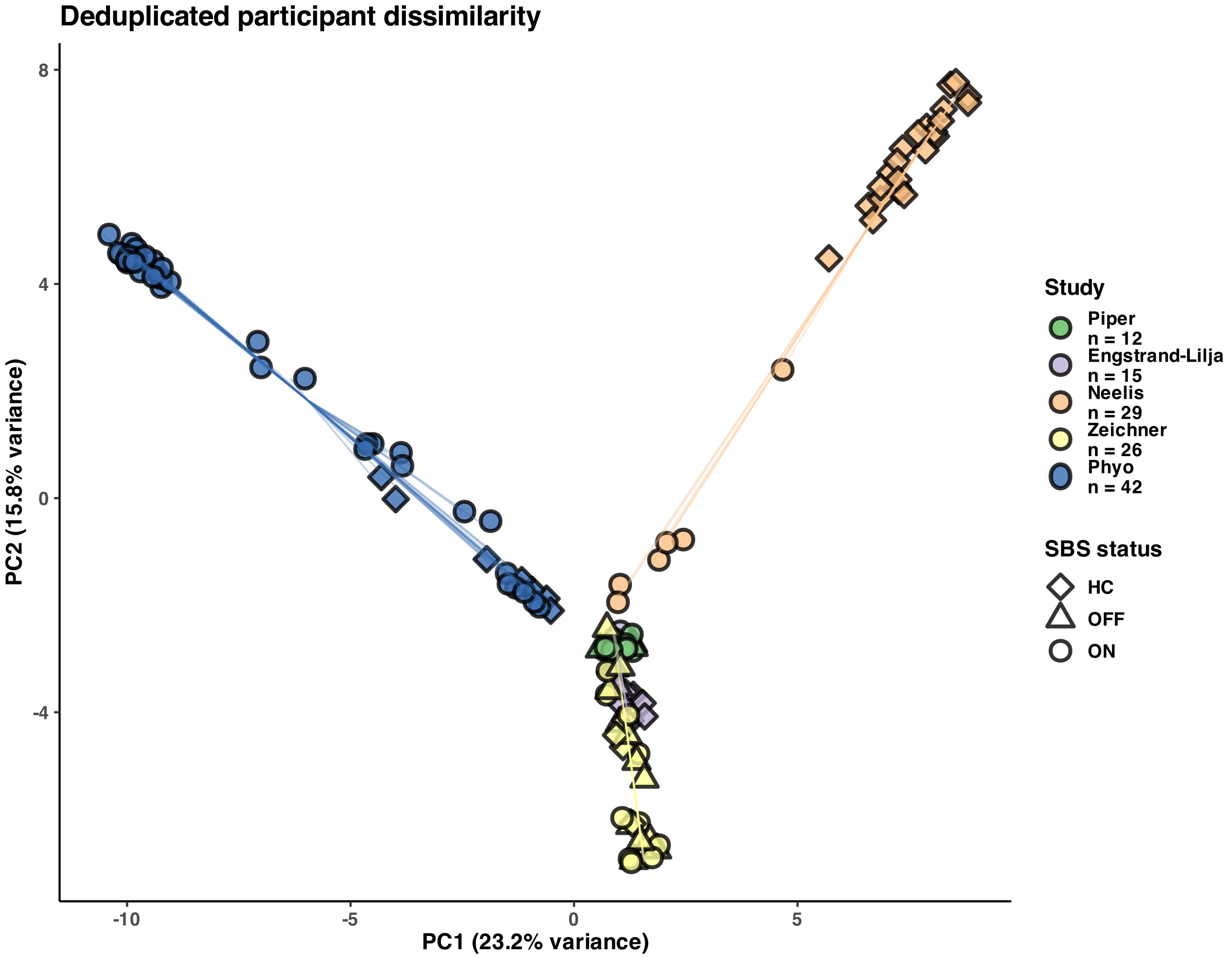

Microbiota composition (weighted Bray-Curtis dissimilarity) of samples included in this analysis. Each point represents an individual sample, coloured by originating study. Sample type is depicted by shape. PC = principle component; SBS = Short-bowel syndrome; HC = Healthy control.

Supplementary figure S4 – Rarefaction depth of 3000 reads is sufficient to characterise microbiota of samples included in this systematic review and meta-analysis

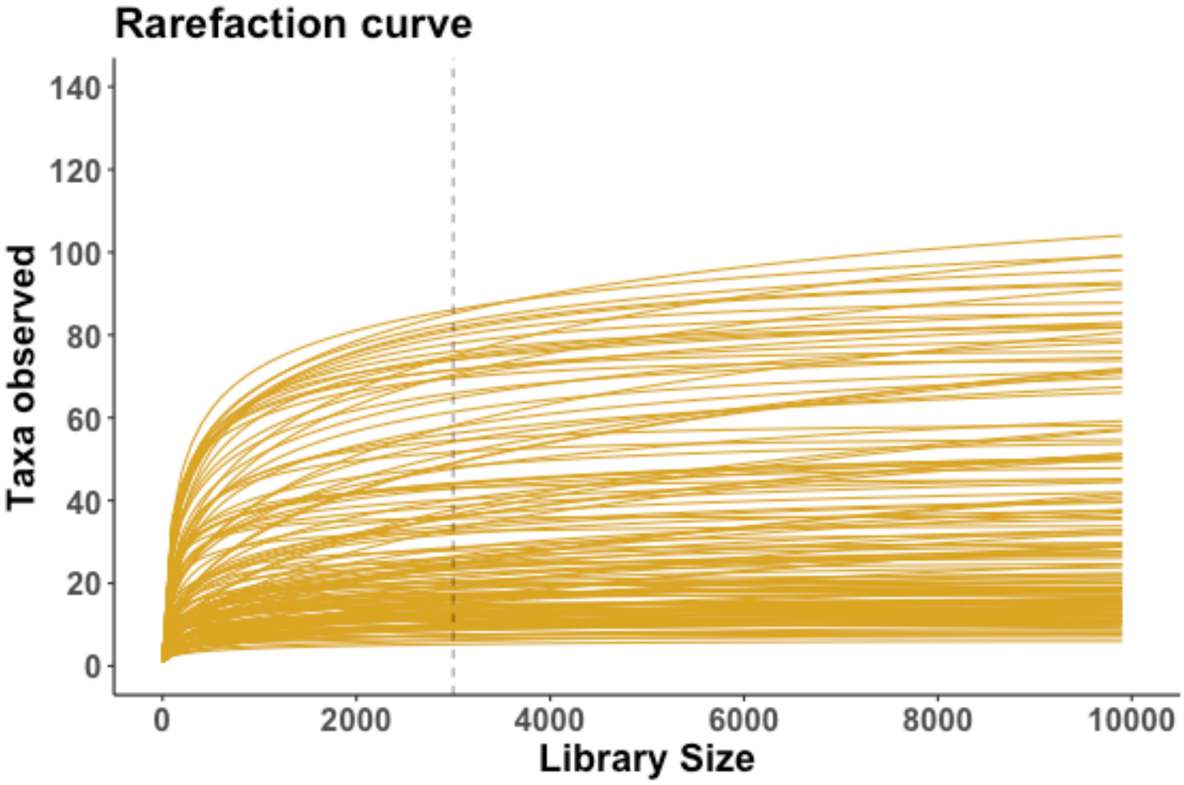

Number of taxa observed with increasing library per sample included in this analysis. Each line represents an individual sample. Dotted line intersecting the x axis represents the rarefaction cut-off of 3000 reads. Sample rarefaction curves have reached asymptote by this point indicating sufficient sampling to characterise microbial populations.

Supplementary table S5 – MaAsLin2 results identify differentially abundant bacterial genera between participants on and off PN as well as between originating studies in this systematic review and meta-analysis

Feature	Variable	value	coef	stderr	N	N.not.0	pval	qval
g__Lactobacillus2	PN	ON PN	0.002	0.001	91	20	0.074	0.192
g__Dorea4	PN	ON PN	-0.007	0.002	91	19	0.000	0.002
g__.Ruminococcus._torques_group4	PN	ON PN	-0.014	0.005	91	20	0.013	0.046
g__Escherichia.Shigella5	PN	ON PN	-0.040	0.020	91	20	0.055	0.154
g__Blautia3	PN	ON PN	-0.067	0.019	91	20	0.001	0.004
g__Escherichia.Shigella3	Study	Phyo	0.558	0.085	91	35	0.000	0.000
g__Escherichia.Shigella2	Study	Zeichner	0.430	0.132	91	17	0.002	0.010
g__Streptococcus2	Study	Zeichner	0.183	0.103	91	19	0.081	0.206
g__Klebsiella2	Study	Phyo	0.107	0.063	91	23	0.093	0.222
g__Staphylococcus3	Study	Phyo	0.055	0.022	91	26	0.019	0.059
g__Bifidobacterium3	Study	Phyo	0.047	0.019	91	31	0.021	0.063
g__Proteus3	Study	Phyo	0.035	0.013	91	20	0.014	0.049
g__Yersinia3	Study	Phyo	0.025	0.014	91	14	0.083	0.206
g__Salinicoccus2	Study	Phyo	0.021	0.009	91	17	0.027	0.078
g__Bacillus3	Study	Phyo	0.003	0.001	91	27	0.001	0.007
g__Haemophilus3	Study	Phyo	0.002	0.001	91	24	0.007	0.029
g__Lactobacillus2	Study	Engstrand -Lilja	-0.003	0.001	91	20	0.012	0.043
g__Lactobacillus2	Study	Zeichner	-0.004	0.001	91	20	0.000	0.002
g__Lactobacillus2	Study	Phyo	-0.005	0.001	91	20	0.000	0.000
g__Lactobacillus2	Study	Neelis	-0.005	0.002	91	20	0.003	0.014
g__Megasphaera4	Study	Phyo	-0.007	0.002	91	20	0.001	0.005
g__Megasphaera4	Study	Neelis	-0.007	0.003	91	20	0.011	0.041
g__Megasphaera4	Study	Zeichner	-0.008	0.002	91	20	0.000	0.001
g__Megasphaera4	Study	Engstrand -Lilja	-0.008	0.002	91	20	0.001	0.007
g__Dorea4	Study	Phyo	-0.008	0.002	91	19	0.001	0.007
g__Dorea4	Study	Neelis	-0.008	0.003	91	19	0.015	0.051
g__Prevotella5	Study	Engstrand -Lilja	-0.009	0.003	91	20	0.005	0.022
g__Prevotella5	Study	Zeichner	-0.010	0.002	91	20	0.000	0.003
g__Prevotella5	Study	Phyo	-0.011	0.003	91	20	0.000	0.001
g__Prevotella5	Study	Neelis	-0.011	0.003	91	20	0.002	0.009
g__Dorea4	Study	Zeichner	-0.012	0.002	91	19	0.000	0.000
g__Dorea4	Study	Engstrand -Lilja	-0.013	0.003	91	19	0.000	0.000
g__.Ruminococcus._torques_group4	Study	Phyo	-0.016	0.006	91	20	0.019	0.059
g__.Ruminococcus._torques_group4	Study	Neelis	-0.016	0.009	91	20	0.084	0.206
g__Bacteroides5	Study	Zeichner	-0.016	0.008	91	20	0.061	0.165
g__.Ruminococcus._gnavus_group5	Study	Zeichner	-0.017	0.009	91	20	0.063	0.169
g__Bacteroides5	Study	Phyo	-0.022	0.008	91	20	0.011	0.041
g__Bacteroides5	Study	Neelis	-0.022	0.013	91	20	0.096	0.225
g__.Ruminococcus._gnavus_group5	Study	Phyo	-0.022	0.009	91	20	0.026	0.078
g__.Ruminococcus._gnavus_group5	Study	Neelis	-0.022	0.014	91	20	0.107	0.248
g__.Ruminococcus._torques_group4	Study	Zeichner	-0.023	0.006	91	20	0.000	0.003

g__.Ruminococcus._torques_group4	Study	Engstrand -Lilja	-0.026	0.008	91	20	0.002	0.009
g__Akkermansia5	Study	Engstrand -Lilja	-0.058	0.034	91	20	0.092	0.222
g__Blautia3	Study	Phyo	-0.058	0.020	91	20	0.006	0.024
g__Blautia3	Study	Neelis	-0.058	0.032	91	20	0.070	0.184
g__Akkermansia5	Study	Zeichner	-0.059	0.025	91	20	0.019	0.059
g__Akkermansia5	Study	Phyo	-0.063	0.025	91	20	0.014	0.049
g__Akkermansia5	Study	Neelis	-0.063	0.039	91	20	0.109	0.248
g__Escherichia.Shigella5	Study	Phyo	-0.073	0.022	91	20	0.002	0.010
g__Escherichia.Shigella5	Study	Neelis	-0.073	0.034	91	20	0.037	0.103
g__Escherichia.Shigella5	Study	Zeichner	-0.094	0.022	91	20	0.000	0.001
g__Blautia3	Study	Zeichner	-0.095	0.020	91	20	0.000	0.000
g__Escherichia.Shigella5	Study	Engstrand -Lilja	-0.103	0.030	91	20	0.001	0.007
g__Blautia3	Study	Engstrand -Lilja	-0.109	0.028	91	20	0.000	0.002
g__Streptococcus5	Study	Engstrand -Lilja	-0.163	0.072	91	20	0.027	0.078
g__Streptococcus5	Study	Zeichner	-0.176	0.058	91	20	0.004	0.017
g__Veillonella3	Study	Phyo	-0.212	0.045	91	20	0.000	0.000
g__Veillonella3	Study	Neelis	-0.212	0.064	91	20	0.001	0.007
g__Streptococcus5	Study	Phyo	-0.213	0.062	91	20	0.001	0.007
g__Streptococcus5	Study	Neelis	-0.213	0.079	91	20	0.009	0.036
g__Veillonella3	Study	Zeichner	-0.222	0.043	91	20	0.000	0.000
g__Veillonella3	Study	Engstrand -Lilja	-0.226	0.057	91	20	0.000	0.002
g__Bifidobacterium5	Study	Engstrand -Lilja	-0.268	0.053	91	20	0.000	0.000
g__Bifidobacterium5	Study	Zeichner	-0.269	0.041	91	20	0.000	0.000
g__Bifidobacterium5	Study	Phyo	-0.272	0.043	91	20	0.000	0.000
g__Bifidobacterium5	Study	Neelis	-0.272	0.059	91	20	0.000	0.000

Coef = coefficient of variation; stderr = standard error; N = number of observations; N.not.0 = number of observations > 0 (taxon is present); pval = Probability value; qval = Adjusted P value (multiple hypothesis testing); PN = Parenteral Nutrition
